# Novel method using hybrid markers: development of an approach for pulmonary measurement of multi-walled carbon nanotubes

**DOI:** 10.1186/1745-6673-8-30

**Published:** 2013-10-25

**Authors:** Makoto Ohnishi, Hirofumi Yajima, Tatsuya Kasai, Yumi Umeda, Masahiro Yamamoto, Seigo Yamamoto, Hirokazu Okuda, Masaaki Suzuki, Tomoshi Nishizawa, Shoji Fukushima

**Affiliations:** 1 Japan Bioassay Research Center, Japan Industrial Safety and Health Association, 2445 Hirasawa, Hadano, Kanagawa 257-0015, Japan; 2 Tokyo University of Science, Department of Applied Chemistry, 1-3 Kagurazaka Shinjuku-Ku, Tokyo 162-8601, Japan; 3 Kanagawa Institute of Technology, Center for Basic Education and Integrated Learning, 1030 Shimo-ogino, Atsugi, Kanagawa 243-0292, Japan

**Keywords:** Multi-walled carbon nanotubes, Novel method using hybrid markers, Fine determination, Rat lungs

## Abstract

**Abstracts:**

**Background**

Multi-walled carbon nanotubes (MWCNT)s are suspected to induce pulmonary and pleural cancers due to their asbestos-like configurations. Therefore, accurate measurement of inhaled nanotubes in target organs is crucial for assessing cancer risk. Conventionally, nanotubes are measured after combustion at high temperature for conversion into CO_2_; however, the sensitivity is poor and the method lacks versatility. We have therefore developed a novel approach using hybrid markers for nanotube analysis, featuring high sensitivity and the capacity to conduct repeated analyses. The method involves adsorption of markers to nanotubes, followed by their desorption and assessment by means of high performance liquid chromatography (HPLC).

**Methods**

Recovery of MWCNT from rat lungs was conducted, and pulmonary MWCNT amounts were determined using rats intratracheally-exposed to MWCNT aerosol at 5 mg/m^3^ for 6 hours/day.

**Results**

The correlation coefficient for the calibration curve of MWCNT weight and the HPLC area was 0.9991. Consequently, the lower quantitation limit yielded was 0.2 μg. The recovery was 92-98% at approximately 0.4-2.0 μg demonstrating that MWCNTs in the lung could be measured accurately and precisely.

**Conclusions**

We have developed a novel method using a hybrid marker approach for nanotube analysis, featuring very high sensitivity and the capacity to conduct repeated analyses. We further confirmed correlations between the amounts of nanotubes and markers and pulmonary nanotube measurement demonstrated that trace amounts could be detected with values closely relating to the administered dose, verifying that the method is sensitive and precise.

## Background

Carbon nanotubes were discovered by Iijima in 1991 [1] and are generally expected to greatly contribute to society because of their structure, size, mass, characteristics as semiconductors, and other electrical properties. However, multi-walled carbon nanotubes (MWCNT)s, due to their fiber-like structure [2], are suspected of causing toxicity resembling that observed with asbestos. In animal experiments, development of mesothelioma of the peritoneum has been reported in mice and rats administered MWCNT intraperitoneally [3,4], and clearly care needs to be taken to avoid adverse human exposure. In addition, CNTs was found to exacerbate murine allergic airway inflammation via enhanced activation of T helper cell immunity and increased oxidative stress [5].

Therefore, accurate measurement of inhaled nanotubes in target organs is crucial for assessing cancer risk. Also, there is a need to investigate possible accumulation of MWCNT in the lungs after entry through the nasal cavity, the anticipated exposure route in human cases [6-8]. The characteristics of MWCNT include insolubility, a fiber-like structure of carbon chains, and indistinctive optical signals (excluding specialized regions). For this reason, quantitative evaluation of MWCNT cannot be performed by the application of general analytical methods [9-11]. Rather reliance has been placed on weighing and carbon analysis, which do not necessarily provide high sensitivity. Conventionally, nanotubes are measured after combustion at high temperature for conversion into CO_2_; however, the sensitivity is poor and the method lacks versatility [12].

Novel methods using hybrid markers are emerging tools for determinations without the need for weight measurement or carbon analysis. Use of a polycyclic aromatic hydrocarbon (PAH) as a marker was reported by Nakashima et al. [13] with adsorption onto MWCNT resulting in a fluorescence quenching effect from the optical perspective [14]; however, investigations have not been conducted on quantitative evaluation of MWCNT by means of adsorbing PAH as a marker, then desorbing and measuring the amount of marker. We have selected benzo[*ghi*]perylene (B(ghi)P) as a marker, for adsorption onto dispersed MWCNT, then desorption using an organic solvent and quantification by HPLC with fluorescence spectroscopy [15,16]. We here document our novel method using the hybrid marker with evidence of its applicability for measurement of MWCNT in the lungs, including after a single exposure in rats.

## Methods

### Test substance

A MWCNT sample was purchased from Hodogaya Chemical, Co. Ltd. (MWNT-7, Lot No. 080126, Tokyo, Japan) and used in the present study as produced; i.e., without being purified or further sieved. Since MWCNTs are not water soluble, the test substance was suspended in 9.6% phosphate-buffered saline containing 0.1% Tween 80 (TW-mixture) as a colloidal dispersant and subjected to ultrasonication for 20 min with an ultrasonic homogenizer (VP-30S, 20 kHz, 300 W, TAITEC Co., Ltd, Tokyo, Japan).

### Animal

Male F344/DuCrlCrlj rats were purchased from Charles River Japan, Inc. (Kanagawa, Japan) at the age of 4 weeks for inhalation exposure and employed at the age of 11 weeks for intratracheal administration and recovery testing. The animals were quarantined and acclimated for 2 weeks, then housed individually in stainless steel wire-mesh hanging cages (170W × 294D × 176H mm) under controlled environmental conditions. For inhalation chambers, the room temperature and the relative humidity were controlled at 23°C ± 2°C and 55% ± 10% with 12 air changes/hour. For intratracheal administration, the room temperature and the relative humidity were controlled at 24°C ± 2°C and 55% ± 10% with 15 to 17 air changes/hour. Fluorescent lighting was controlled automatically to provide a 12-hour light/dark cycle. All rats had free access to sterilized water and γ-irradiation-sterilized commercial pellet diet (CRF-1, Oriental Yeast Co., Ltd., Tokyo, Japan). The animals were cared for in accordance with the Guide for the Care and Use of Laboratory Animals [17], and the present study was approved by the ethics committee of the Japan Bioassay Research Center (JBRC).

### Recovery test design

To validate the proposed method, a recovery test was performed by spiking lung tissue. A total of 5 rats were employed, and their lungs were removed for the recovery test. The mean and standard deviation (SD) of body weights were 253.1 ± 12.4 g at the commencement. After inhalational anesthetization with isoflurane gas (Forane, Abbott Japan Co., Ltd., Tokyo, Japan), all rats underwent complete necropsy, when lungs were removed and weighed. The lungs were fixed in 10% neutral buffered formalin at 1 week. Then, a TW-mixture solution containing 2 μg of MWCNT was added and the lungs were measured for MWCNTs as detailed below.

### Intratracheal administration test design

A total of 5 rats were used for the intratracheal administration of MWCNT. The initial body weight mean and SD were 262.2 ± 11.7 g. Before intratracheal administration, the ultrasonicated suspension of MWCNTs was further subjected to additional ultrasonication for 30s with a sonicator (US-2, AS ONE Co., Ltd., Tokyo, Japan). After inhalational anesthetization with isoflurane gas (Forane, Abbott Japan Co., Ltd., Tokyo, Japan), the suspension of MWCNT in TW-mixture (0.3 ml) was intratracheally administrated [18], using a microspray cannula of an Intratracheal Aerosolizer (1A-1B, PennCentury, Inc., USA). Rats received MWCNTs at a dose of 2 μg/animal. At subsequent complete necropsy, the lungs and trachea were removed, weighed, and fixed in 10% neutral buffered formalin for 1 week. Then the lungs were assessed for MWCNTs by the method detailed below.

### Inhalation exposure test design

Aerosol generation and whole body inhalation exposure to MWCNTs: The system and method for generation of MWCNT aerosols and inhalation exposure of rats to a dry aerosol in chambers were described in detail earlier [19]. Twenty five rats were exposed to MWCNT aerosol at a target concentration of 5 mg/m^3^ for 6 hours/day. The mean and SD of body weights were 123.3 ± 6.1 g for all rats. At the end of the six-hour exposure period, anesthetization with isoflurane gas was performed for 5 rats for necropsy, and their left lungs were removed, weighed, fixed in 10% neutral buffered formalin for 1 week and employed for measurement of MWCNT as detailed below. Groups of five remaining animals were necropsied on 1, 7, 28 or 56 days after the exposure for determination of time change in MWCNT deposits.

### Sample preparation for generation of a calibration curve

A 10 mg sample of MWCNT was added to 40 ml of TW-mixture and then sonicated during a 30-min cooling period. The solution was diluted to 2 μg/ml with C99, then 0.4, 0.8, 1.2, 1.6 μg/ml C99 additional solutions were prepared as further standards, 0.1 ml of each being used for analysis. The standard solutions were centrifuged (12000 rpm, 10 min), and the supernatants were removed and after addition of 0.5 ml of TW-mixture were stirred and centrifuged.

### Acidic preparation

Following removal of the supernatants and addition of 100 μl concentrated sulfuric acid, the resultant solution was stirred and MWCNTs adhering to Nuclepore membrane filters (Whatman; 111109, pore size; 0.8 μm, diameter; 47 mm) were extracted under ultrasound with 1 ml of TW-mixture and 0.5 ml aliquots of MWCNT solution were transferred to glass tubes for HPLC autosample analysis.

### Lung digestion

**Figure 1:**
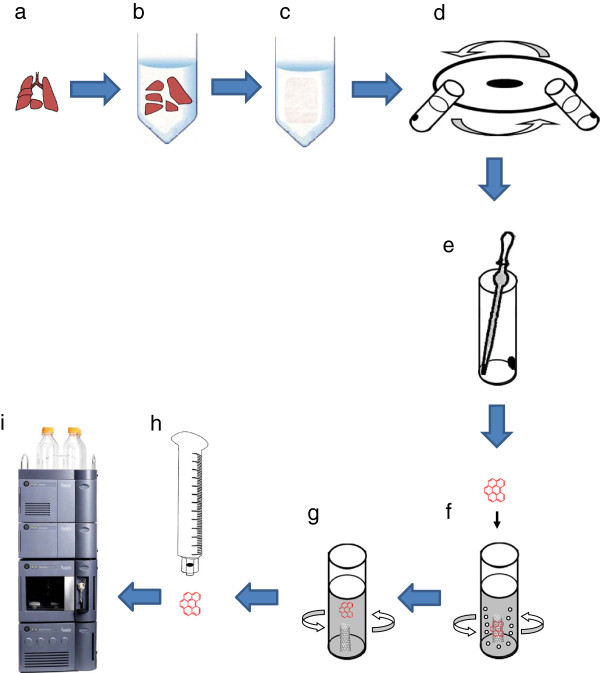
**Novel method using hybrid markers for MWCNT. a**, Lung exposed to MWCNT. **b**, Transfer of lung tissue into C99 at room temperature. **c**, Lung digestion in C99. **d**, Lung solution centrifugation at 12,000 rpm for 10 minutes. **e**, Supernatant removal. **f**, Novel method using hybrid markers: Marker adsorbtion onto MWCNT for 15 min. **g**, Extraction of adsorbed B(ghi)P on MWCNTs to acetonitrile. **h**, Filtration of B(ghi)P in the MWCNT. **i**, HPLC analysis.

After fixation in 10% neutral buffered formalin for 1 week, lung samples (Figure 1a) were allowed to react with C99 at room temperature overnight [20] (Figure 1b). The digested solution (Figure 1c) was then centrifuged at 12,000 rpm for 10 minutes (Figure 1d) and the supernatant was removed (Figure 1e). A 0.5 ml aliquot of TW-mixture was added followed by stirring and further centrifugation and then “acidic preparation” and “novel method using hybrid markers and HPLC analysis” were performed as described below.

### Novel method using hybrid markers and HPLC analysis

After adding 0.125 μg/ml B(ghi)P and 25 μl acetonitrile solution (Figure 1f), the solutions were stirred for 15 min to produce MWCNT solutions with actual concentrations of 0.2, 0.4, 0.6, 0.8, and 1.0 μg/ml. The solutions were then centrifuged, and the supernatants were removed. After addition of 0.5 ml D.W., the resultant solutions were stirred and then centrifuged again. After removal of the supernatants, addition of 0.5 ml acetonitrile, and stirring (Figure 1g), the adsorbed B(ghi)P in MWCNTs was extracted to acetonitrile and filtered (Figure 1h) for HPLC analysis. Chromatography was performed using an Acquity UPLC system (Waters, Milford, MA, U.S.A.) coupled to a fluorescence detector FLR (Waters). Eluates were analyzed quantitatively by monitoring at fluorescent wavelengths of 294 nm for excitation and 410 nm for emission with 5 ml aliquots of extract injected onto a 1.7 mm C18 100 × 2.1 mm I.D. Acquity BEH column (Waters) (Figure 1i). The mobile phases were acetonitrile : methanol : distilled water =75 : 20 : 5. The peak of the injected sample was detected at about 1.3 min. An eluent flow rate of 0.5 ml/min was used for all analyses.

## Results and discussion

**Figure 2:**
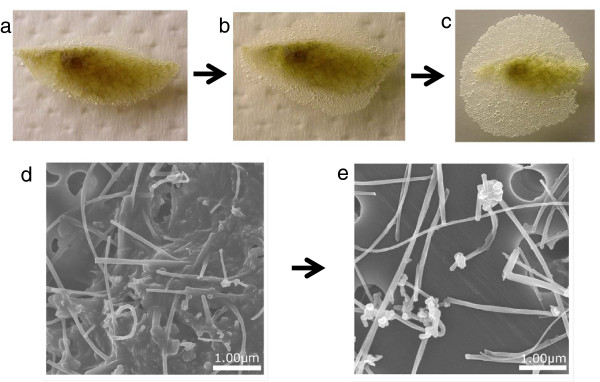
**Sample preparation for MWCNT.** The lungs were dissolved with laboratory bleach (Clean 99-K200^R^) at room temperature overnight. **a**, Sample immediately after starting lung dissolution. **b**, after 5 min. **c**, after 10 min. SEM images of MWCNTs centrifugally sedimented in the lung-dissolved solution. **d**, Before treatment with sulfuric acid. **e**, After treatment with sulfuric acid.

By treating the lungs removed from rats with formalin solution (10% neutral buffered formalin) and Clean 99-K200^R^ (C99) in advance, the lungs could be rapidly dissolved (within 30 min) (Figure 2a,b and c). With regard to the analytical method, it was clear that for extraction of MWCNT from the lungs, dissolution with C99 is markedly faster after immersion in formalin compared to dissolution with C99 only. Scanning electron microscope (SEM) observation of centrifuge sediments of lung-dissolved solution, however, revealed MWCNT surrounded by undissolved components (Figure 2d), mainly connective tissue. The addition of concentrated sulfuric acid to the sediment led to the complete dissolution of sediment components other than MWCNTs, and as a result of subsequent filtration, MWCNTs were isolated as the only residue (Figure 2e).

**Figure 3:**
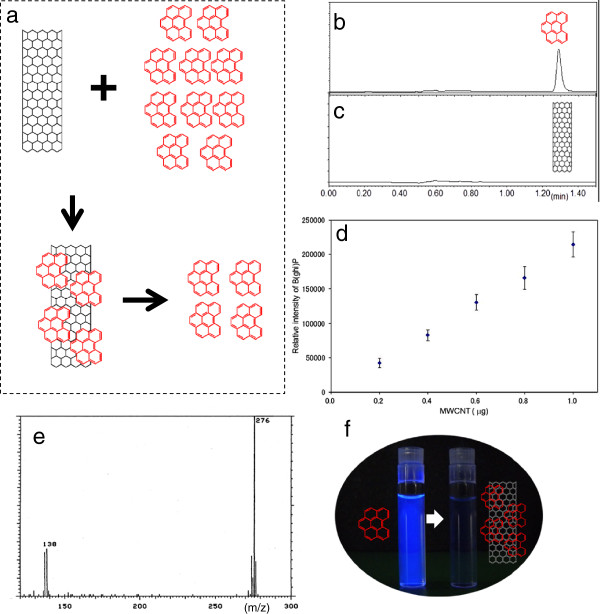
**Adsorption and desorption of the marker (B(ghi)P). a**, Processes of adsorbtion onto the surface of the MWCNT and desorption with organic solvent. **b**, HPLC chromatogram of MWCNTs with markers. **c**, HPLC chromatogram of MWCNT without markers. **d**, Calibration curve of MWCNT. **e**, GC-MS mass spectrum of MWCNT with the marker. **f**, Quenching effects of MWCNT with marker adsorption.

MWCNTs, with clean smooth surfaces due to alkali (C99) and acid (sulfuric acid) treatments, were dispersed by ultrasonic vibration, and then B(ghi)P was adsorbed for 15 min, followed by centrifugation and removal of the supernatant. Finally, the marker was desorbed with acetonitrile in organic solvent (Figure 3a), and examined by HPLC. Chromatograms of MWCNT with B(ghi)P revealed a peak at 1.3 min (Figure 3b), no peak being observed with MWCNT alone (Figure 3c); this confirmed that untreated MWCNT had no B(ghi)P on their surfaces. Ten measurements were made to generate a calibration curve of MWCNT, the relationship between the concentration and area being shown in Figure 3d. Repeated generation of calibration curves using this method gave consistently similar values. The lower quantitation limit yielded was 0.2 μg. The correlation coefficient was 0.9991, confirming the linearity and reliability of the calibration curve. To confirm that B(ghi)P was adsorbed onto the surfaces of MWCNT, B(ghi)P-adsorbed MWCNT were directly introduced into a mass spectrometer, and the mass spectrometry measurement revealed peaks at m/z = 276 and m/z = 138, coincident with the molecular ion peak (m/z = 276) and a fragment peak (m/z = 138) of B(ghi)P (Figure 3e). Pale-blue fluorescence was observed upon UV lamp irradiation of B(ghi)P dissolved in TW solution while such fluorescence was not seen with B(ghi)P solution containing MWCNTs, due to the quenching effects of B(ghi)P-adsorbed MWCNT (Figure 3f). In addition, after addition of a defined amount of MWCNT to rat lungs removed in advance, collection rates obtained according to the analytical procedure above confirmed spiked recovery, measurement accuracy, and repeatability.

**Figure 4:**
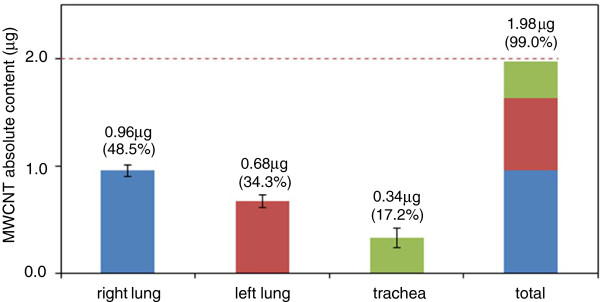
**Measurement of MWCNT in the lungs of rats administered by the intra-tracheal route.** Values are mean ± SD.

Recovery was 92.5% at approximately 0.4 μg, 93.0% at 1.0 μg, and 98.0% at 2.0 μg, demonstrating that MWCNTs in the lung could be measured accurately and precisely. Furthermore, upon intratracheal administration of 2 μg MWCNT to rats and subsequent measurement of MWCNT with this method, 0.68 μg (34.3%), 0.96 μg (48.5%), and 0.34 μg (17.2%) of MWCNT were collected from the right lung, left lung, and trachea, respectively (Figure 4). Although it was possible that MWCNTs remained in the trachea, the results showed that almost all the administered tubules could be recovered from the lungs and the trachea and that the amount could be appropriately measured despite a very low dose. The fact that the right lung was found to contain more MWCNTs is consistent with the larger size as compared to the left lung.

**Figure 5:**
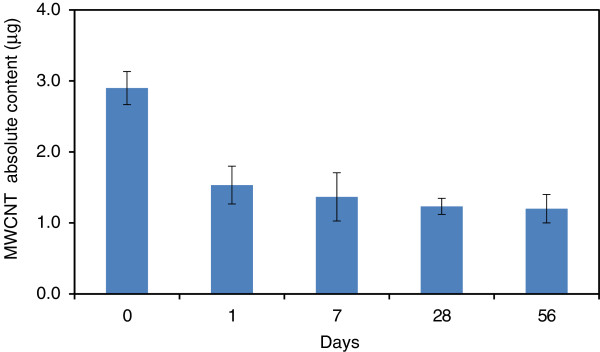
**Time-course changes in the amount of MWCNT in the lungs of rats after inhalation exposure.** Values are mean ± SD.

Following the method of Kasai et al. [19], when rats were exposed to MWCNTs by whole body inhalation exposure, the amounts of tubules decreased to approximately one half on day 1 of the post-exposure period, with subsequent further gradually decrease on days 7, 28, and 56 (Figure 5). As a result of measuring the amount of MWCNT in the left lung of rats after a single inhalation exposure, the absolute amount was actually found to be not more than 2 μg [19]. With the method of Tamura et al. [12], the lower quantitation limit of MWCNT was thought to be 1 μg, in contrast to the 0.2 μg established here.

In the future, correlation studies of inhaled CNTs in the lung and lung inflammatory status should be conducted as this is an important issue for which we could utilize this method in environmental toxicology.

## Conclusions

In conclusion, our novel method using hybrid markers offers a new approach for very accurate measurement of multi-walled carbon nanotubes. Because of the nature of the adsorption and desorption processes, weights of MWCNTs and marker levels directly correlate. Furthermore, our method here proved applicable to measurement of nanotubes in lungs of animals after administration in in vitro/in vivo models. The novel method using hybrid markers provides a platform to study MWCNTs with high sensitivity and versatility, with the ability to conduct repeated analyses. This technique should facilitate assessment of nano-bio interactions and carcinogenicity of nanotubes. Since the socioeconomic potential of MWCNT is two-sided (with both benefit and risk), comprehensive safety studies need to be conducted. For this reason, there is a further need to investigate the amounts of MWCNT accumulating in the lungs, the organ most impacted by inhalation of nanotubules.

## Abbreviations

B(ghi)P: Benzo[*ghi*]perylene; C99: Clean 99-K200^R^; HPLC: High performance liquid chromatography; MWCNT: Multi-walled carbon nanotube; PAH: Polycyclic aromatic hydrocarbon; TW-mixture: 9.6% phosphate-buffered saline containing 0.1% Tween 80; SD: Standard deviation; SEM: Scanning electron microscope.

## Competing interests

The authors declare that they have no competing interest.

## Authors’ contributions

MO and HY conceived and designed the experiments. MO, MY and MS performed the experiments. MO, TK, YU, SY and TN analysed the data. HO contributed toxicological information. MO and SF co-wrote the paper. All authors discussed the results and commented on the manuscript. All authors read and approved the final manuscript.
